# Fractures of the proximal radius in children: management and results of 100 consecutive cases

**DOI:** 10.1007/s00402-021-03917-w

**Published:** 2021-05-11

**Authors:** Markus Dietzel, Simon Scherer, Michael Esser, Hans-Joachim Kirschner, Jörg Fuchs, Justus Lieber

**Affiliations:** 1 Department of Pediatric Surgery and Pediatric Urology, University Children’s Hospital, Hoppe-Seyler-Strasse 3, 72076 Tübingen, Germany; 2 Department of Diagnostic Radiology, University Hospital, Hoppe-Seyler-Strasse 3, 72076 Tübingen, Germany

**Keywords:** Pediatric trauma, Radial neck fracture, Proximal radius fracture, ESIN, Elastic stable intramedullary nailing, Pseudarthrosis

## Abstract

**Introduction:**

Pediatric radial neck and head fractures are rare, accounting for only 1% of all fractures in children. The aim of this study is to describe the management and results of the respective fracture types and different injury characteristics.

**Materials and methods:**

This study performs a retrospective data analysis of 100 consecutive patients with a fracture of the proximal radius treated in a single high-volume pediatric trauma center.

**Results:**

One hundred patients [mean age 7.5 years (1–15)] were documented with a fracture of the proximal radius between 3/2011 and 12/2019. The gender distribution was 62 girls and 38 boys. Twenty-seven patients had concomitant injuries. Conservative treatment was performed in 63 patients (Judet I = 27; II = 30; III = 6; Mason I = 2) using an above-the-elbow cast for 21 days (6–35). Surgical treatment was performed in 37 patients (Judet II = 3; III = 22; IV = 5; V = 7) using elastic stable intramedullary nailing (ESIN). Open reduction was necessary in five cases, and additional immobilization was performed in 32 cases. Six complications occurred: loss of implant stability (*n* = 2), healing in malalignment, pseudarthrosis, radioulnar synostosis, and a persisting hypoesthesia at the thumb. As a result, two ESIN osteosynthesis were revised, and one radial head resection was performed. Loss of movement was seen in 11% of cases, overall Mayo elbow performance index (MEPI) was 99.8 (90–100), and none of the patients experienced negative impacts on activities of daily life.

**Conclusions:**

Proximal radial fractures occur predominately without dislocation. Good results are obtained with conservative treatment throughout. In cases with displacement exceeding growth-related correction, ESIN is the undisputed treatment of choice. Open surgery and long immobilization periods should be avoided whenever possible.

## Background

Fractures of the proximal radius account for only 1% of all fractures and from 4.5 to 21% of elbow fractures in children [1–3]. Almost all cases involve radial neck fractures (RNF), and radial head fractures (RHF) represent a rarity in this group. The most common injury mechanism is a fall on the outstretched arm with the forearm in supination and an associated valgus thrust. Another possible mechanism, although rare, is a fall on the hyperextended elbow with the forearm in pronounced pronation. Both mechanisms cause compression of the radiocapitellar joint [4]. RNF are predominant among children aged 8–12 years. Associated fractures of the olecranon, medial epicondyle, or lateral condyle occur in up to 50% of cases [5]. The high potential of spontaneous correction and the vascular supply of the proximal radius make the decision for the respective treatment method demanding. The results after RNF are predominantly described as good in the literature for surgical and conservative concepts [6–8]. However, some authors mention complication rates of up to 27–37% [9, 10]. This dissent may be explained by several points. The potential of growth-related correction is unknown, and the exact mechanisms of remodeling are not fully understood. As a result, overtreatment is an issue in some patients. Also, the increasing popularity of intramedullary fixation may cause a trend toward surgical interventions as previously described for other pediatric fractures [11]. In addition, surgical methods and postoperative treatment are not yet standardized and may lead to the application of disproportionately invasive methods.

Different fracture types, various additional injuries, and a large age range of the patients make a uniform evaluation difficult, while the scientific research concentrates on highly specific issues. Therefore, the aim of this study is to describe a single-center experience regarding the treatment of a consecutive group of patients with proximal radius fractures. In particular, this study focuses on the different fracture types, pitfalls of treatment modalities, and final results.

## Materials and methods

### Patients and ethical considerations

This study retrospectively analyses the charts of 100 consecutive patients below 16 years of age with a fracture of the proximal radius treated at our institution between March 2011 and December 2019. The authors used clinical charts to collect demographic characteristics, clinical backgrounds, indications for operation, treatment procedures, complications, and postoperative outcomes including radiographic findings and clinical examination results. The data were stored on a computerized database. The data were also acquired and processed according to the latest version of the “World Medical Association Declaration of Helsinki—Ethical Principles for Medical Research Involving Human Subjects”. This study was approved by the local ethical committee (project no. 079/2020BO2).

Conservative treatment refers to immobilization in an above-the-elbow cast. In cases with a potential risk for secondary dislocation, conventional radiographic controls were performed. Surgical intervention was indicated for one of the following three occasions: (1) when the proximal radius was completely displaced; (2) when the radial neck showed an angulation exceeding 45° in children below 10 years of age or 20° in children above 10 years of age; or (3) when an articular step or gap > 2 mm was seen in radial head fractures. The concept of surgical treatment foresaw ESIN osteosynthesis in fractures of the radial neck or proximal radius and mini screw osteosynthesis in cases of displaced radial head fractures. Additional immobilization was not obligatory. Concomitant injuries were treated on an individual basis. The therapy regimen has been left unchanged throughout the observation period. Complications were classified as proposed by Dindo and Clavien [12].

All patients received follow-up in the outpatient clinic with a range of movement measurement (neutral zero method) after a warmup exercise. We considered normal range of motion (ROM) from 0° to 145° of elbow extension and flexion and from 0° to 70° of forearm pro/supination. Control radiographs were individually performed to detect growth disturbances and measure axial deviations. For functional assessment, the Mayo elbow performance index (MEPI) [13] was used. The outcome was considered excellent when patients were free of pain and had no limitation (LOM) of elbow extension/flexion and forearm pro-/supination on clinical examination. The results were good or fair in patients with mild or moderate LOM less than 10° or between 10 and 20° and without impairment in daily activities. However, they may experience temporary discomfort during sport activities. The results were poor in children with persisting pain and severe deficits of movement during sport and activities of daily life (LOM > 20°).

### Statistics

This study performs statistical analysis of the different groups using students’ *t* tests and through an analysis of variance with Microsoft Excel (www.microsoft.com). *p* < 0.05 is considered statistically significant.

## Results

**Fig. 1:**
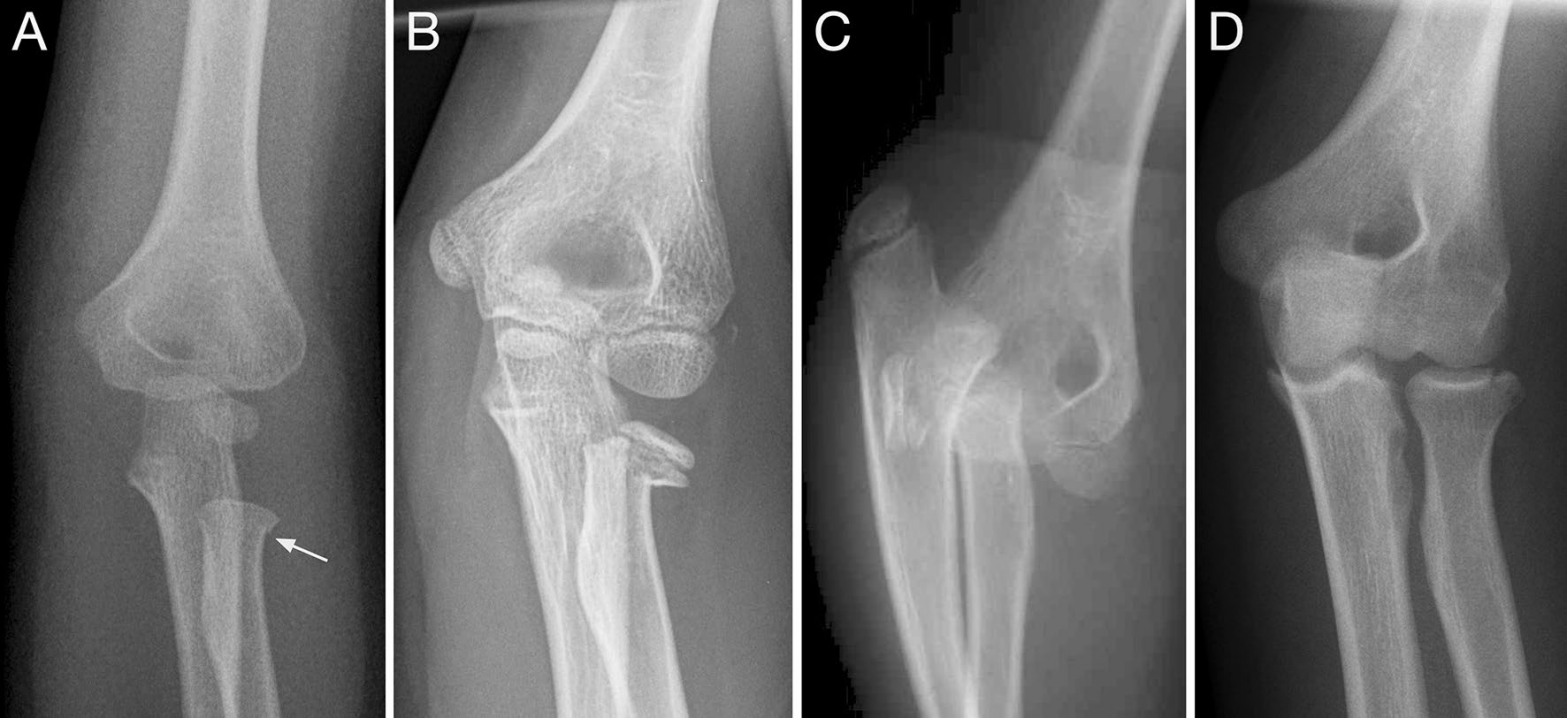
Different types of proximal radius fractures representative for different dimensions of stability, courses and prognosis. Stable buckle fracture of the radial neck (→ arrow) in a 4-year-old girl (**a**). Salter–Harris II fracture of the radial neck with the risk of further dislocation (**b**). Complete dislocation of a metaphyseal radial neck fracture in a 12-year-old boy with elbow dislocation, presenting the worst prognosis due to the complete disruption of the nutritive vessels possibly resulting in total or partial necrosis or pseudarthrosis (**c**). Intraarticular fracture of the radial head representing an adulthood fracture in a 15-year-old adolescent boy with closed physis (**d**)

One hundred patients were documented with a fracture of the radial neck (*n* = 98) or head (*n* = 2) between 3/2011 and 12/2019. The mean age was 7.5 years (1–15 years). The gender distribution was 62 girls and 38 boys. One patient sustained radial neck buckle fractures of both arms simultaneously, and one child suffered from a new fracture one year after the first injury (both non-displaced). Table 1 lists all classified fractures, as proposed by Judet [14] and Mason [15]. Figure 1 shows examples of various fracture types of the proximal radius. In terms of fracture type distribution, the most common type was metaphyseal radial neck fractures including Harris–Salter type I and II lesions (46%), followed by stable buckle fractures (19%). 34 fractures were non-displaced, 31 fractures showed a mean axial deviation of 20.7° (3°–50°), and 8 fractures were completely displaced with no contact of the proximal radius to the shaft. The remaining 27 cases had additional elbow injuries, which are listed in Table 3.

**Table 1:** Classification and management of 100 consecutive fractures of the proximal radius

Management Classification	Conservative treatment (immobilization; *n* = 63)	Surgical treatment (ESIN; *n* = 37)
Judet I	25	–
Judet II	30	3
Judet III	6	22
Judet IV	–	5
Judet V	–	7
Mason I	2	

Judet classification of 98 radial neck fractures, and Mason classification of 2 radial head fractures. Conservative treatment consisted of immobilization in an above-the-elbow cast; surgical treatment consisted of ESIN-osteosynthesis

Conservative treatment was performed in 63 patients (Judet I = 25; II = 30; III = 6 and Mason I = 2) for a mean of 21 days (6–35). Operative treatment was performed in 37 patients (Judet II = 3; III = 22; IV = 5; V = 7). Patients with a Judet type II or III injury were thus treated either surgically or conservatively, according to their age. In all surgically treated cases, ESIN osteosynthesis was performed (Table 1). Additional open reduction for completely displaced fractures was necessary in 5 cases. Additional plaster immobilization was performed in 32 of 37 operated cases either due to additional injuries (Table 2), or in short term for analgetic reasons. Two patients underwent physiotherapeutic treatment after metal removal.

**Table 2:** Treatment and results of 100 consecutive fractures of the proximal radius

	Conservative treatment (cast immobilization)	Surgical treatment (ESIN osteosynthesis)
*n* =	63	37
Age (years)	7.2 (1–15)	8.1 (4–12)
Additional injuries	16 olecranon fractures 1 ulna shaft fracture 1 medial epicondyle fracture	5 olecranon fractures 1 ulna shaft fracture 1 medial epicondyle fracture 3 elbow dislocations
Additional surgical procedures	1 epicondylar screw^a^	4 olecranon wiring 1 olecranon screw 1 ulna shaft ESIN 1 epicondylar screw
Open reduction (*n*)		5
Additional immobilization (*n*)		32
Immobilization period (days)	21 (6–35)	21 (1–46)
Time to implant removal (days)		90 (33–268)
Complications	–	2 loss of implant stability (IIIb)^a^ 1 radioulnar synostosis (IIIb) 1 pseudarthrosis (IIIb) 1 healing in malalignment (I) 1 hypoesthesia at the thumb (I)
Outcome		
LOM	6	3
ADL complaints	–	–
MEPI	99.6 (90–100)	100

Treatment course and results after conservative and surgical treatment of proximal radius fractures in 100 children. Complications were graded according to the classification proposed by Dindo & Clavien [11]. Outcome was determined by the rate of loss of elbow movement (LOM), impairment of activities in daily life (ADL), and MEPI (Mayo elbow performance index)

^a^Screw fixation of an epicondylar avulsion fracture which occurred simultaneously to the (conservatively treated) radial neck fracture

In total, six complications occurred consisting of loss of implant stability (*n* = 2), healing in malalignment (*n* = 1), radioulnar synostosis (*n* = 1), pseudarthrosis (*n* = 1), and a persisting hyposensibility at the thumb (*n* = 1). Consequently, two ESIN osteosynthesis were revised, and one radial head resection was performed. The latter was indicated due to radioulnar synostosis and radial neck pseudarthrosis with consecutive limitation of forearm rotation.

Patients treated with ESIN started spontaneous elbow mobilization after a mean of 21 days (1–46). In surgically treated cases, ESIN removal was performed after a mean of 90 days (33–268).

The mean follow-up of all patients was 6.3 months (1–48) with significantly longer observation period in patients with severe displacement or additional injuries (*p* = 0.008, Table 3). For the last control, 11 patients showed persistent reduction of elbow/forearm movement, but no patient voiced complaints regarding their daily routine. The outcome according to the Mayo elbow performance score [13] was > 90 points in all patients, leading to an “excellent” rating (Table 2). Regarding the different fracture types, excellent results were found in stable and/or non-displaced fractures (Table 3). Excellent and good long-term results were reported in the group with displaced fractures and/or additional injuries. Surprisingly, excellent results were also observed in cases with completely displaced fractures. However, these results were obtained after surgical treatment of the abovementioned complications had been performed. The case in which a radial head resection had been performed also yielded excellent results with a Mayo rating of 100.

**Table 3:** Outcome of proximal radius fractures considering the initial fracture type

	buckle fracture (Judet I)	SH I + II fractures (Judet I/II/III)	RNF with complete displacement (Judet IV)	RNF with additional injuries^a^ (Judet I–IV)	*p*
*n* =	19	44	8	27	
Age (years)	8.2	8.1	5.9	6.6	0.05
Displacement					
None	19	15			
Axial deviation		31 (20.7°)			
No contact			8		
Physiotherapy	–	1	–	1	
Follow-up (months)	3.7	5.2	6.4	10	0.008
LOM					
Mild	1	6		2	
Moderate		1			
Severe		1			
MEPI	100	99.6	100	99.8	

Outcome depending on the respective fracture types (SH = Salter–Harris). Two radial head fractures (Mason I) were excluded from this table due to the small group size. Additional injuries (^a^) were other fractures of the upper extremity and/or elbow dislocations, respectively. Comparing these groups, significant differences were found in follow-up times

## Discussion

This study analyzes data from different types and severity grades of proximal radius fractures, including patients with additional injuries (Fig. 1). The heterogeneity of the cohort was accepted to illuminate the full spectrum of this fracture. We observe that non-displaced Salter–Harris type I and II and stable buckle fractures are the most common forms of injury and that conservative treatment consistently leads to excellent outcomes. Additional fractures occurred in 27% of cases. Three percent of those cases involved accompanying lesions with elbow dislocations, which led us to suspect that the rate of poor outcomes increases with injury severity. In comparison with data from the literature, this study finds similarities regarding mean age and distribution of fracture types. This indicates that we collected a representative cohort of patients [1–3]. This also holds true for the gender distribution of this fracture type, as girls were affected more often. Kang et al. notes that the pre-trauma elbow carrying angle with its greater valgus alignment in girls is one possible explanation [1].

**Fig. 2:**
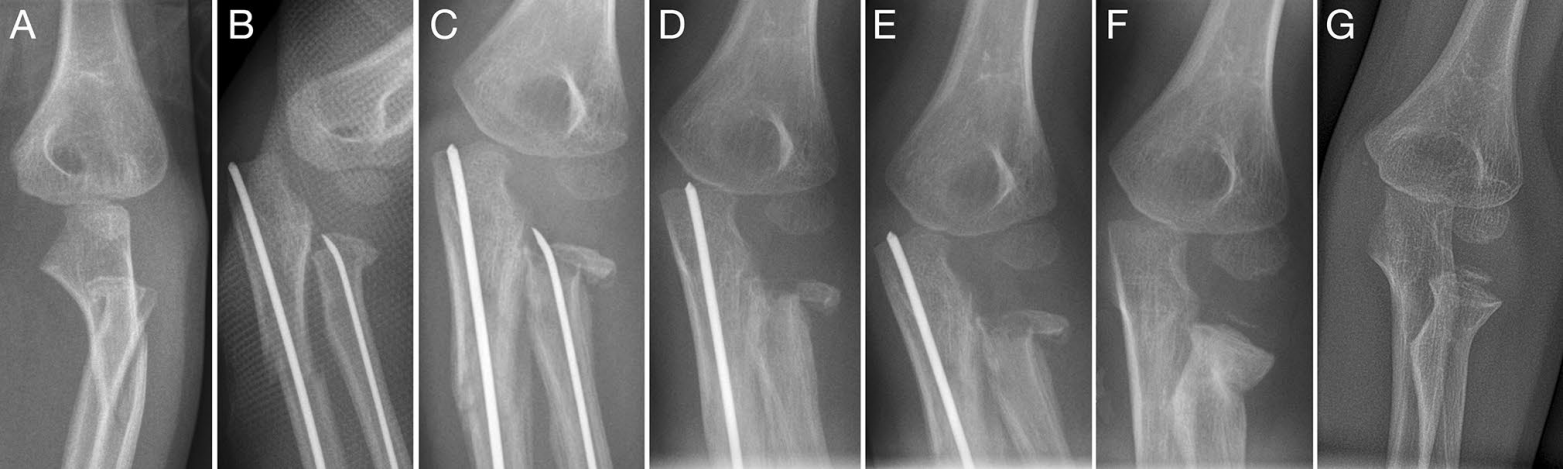
Complete fracture of the metaphyseal radial neck and proximal ulna shaft fracture in a 5-year-old boy treated with ESIN (**a**). The postoperative control showed axial alignment of both fractures (**b**). Consolidation was documented after four weeks, but the radial implant showed evidently missing the radial head, which represents a technical complication that should not have been overlooked intraoperatively (**c**). Due to the consolidation process and to not further damage the blood supply, we refrained from a nail revision and the nail was removed consecutively (**d**). Following this, pseudarthrosis seemed to develop, but complete consolidation (**e**) and remodeling were observed after 11 months (**f**), and 2 years (**g**). In this age group, an enormous potential for growth-related correction exists, however, it should never be overestimated

In RNFs, treatment decisions should consider the growth-related potential of correction. If additional injuries occur, an individual approach should be chosen [7]. Most authors advocate the reduction of radial neck fractures angulated more than 30°, although other authors tolerate 45° [16], which we share in this study. Moreover, an initial angulation of 60° was observed to remodel spontaneously in children below 10 years of age [8]. However, the exact potential of growth-related correction and the mechanisms responsible are still unknown. They cannot be verified more precisely because a respective analysis would be associated with relevant methodological problems and thus would be unrealistic: In a prospective randomized trial, a foreseeable treatment intensification (e.g., AFIC study, trial registration: DRKS00004874) would have to be accepted for reasons of scientifically studying the issue [17]. Consequently, overtreatment might occur, which is also highlighted for other pediatric fractures, especially those of the forearm [18]. The underestimation of the potential of growth-related correction is associated with a higher rate of surgical interventions. This, in turn, may then be associated with the occurrence of complications (Fig. 2). Therefore, it is imperative that the growth-related potential of correction should be included in any treatment plan. However, this potential has to be determined individually for every patient, considering their age, the amount of displacement, and additional injuries. Adherence to actual pediatric trauma guidelines and concepts should be corner stone of treatment in affected patients rather than simply adjusting concepts from adult traumatology onto injured children.

In contrast, the presented cohort also includes movement restrictions after tolerably displaced and conservatively treated fractures. This is important given that the rate of conservative therapy (63%) considerably outweighs that of surgical treatment (37%). The reasons for the movement restrictions in this group are not obvious. In principle, atraumatic treatment is indicated for all proximal radius fractures. This includes short immobilization periods and early functional mobilization, and these have a positive effect on the final functional results after both surgical and conservative therapy, even if the mechanisms are still unknown [19]. However, in our collective, the mean immobilization time of 21 days was too long and was justified only in cases with additional injuries or pronounced soft tissue afflictions. In children without additional injuries, internal processes were responsible for a prolonged immobilization. In the future, an accurate selection must be made according to the fracture type, its stability criteria, and the patient’s age to integrate the earliest possible movement into the treatment concept. Physiotherapy, which can lead to poor functional outcomes if used excessively [20], was only used in two cases in our cohort and thereby does not appear to be a decisive factor. However, physiotherapy may be justified in older children and adolescents, but this also requires prospective scientific evaluation in addition to specific selection criteria.

**Fig. 3:**
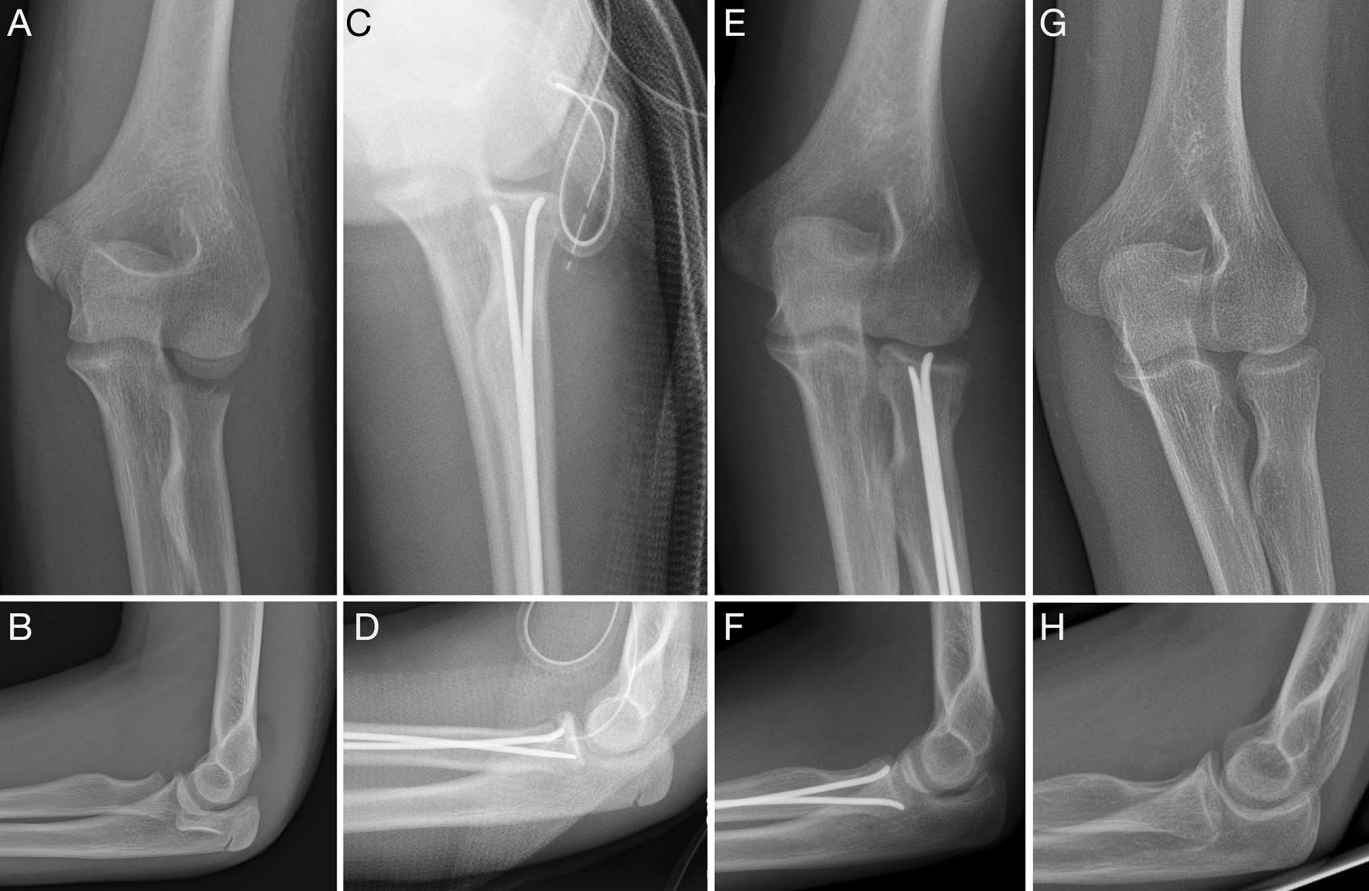
Completely displaced proximal radial fracture in a seven-year-old girl (**a**, **b**). Open reduction and stabilization were performed using two ESIN implants to provide maximal stability to the radial head, which healed even though having been totally deperiostized (**c**, **d**)

If operative treatment is necessary, ESIN is the method of choice throughout the literature [6, 7, 9]. This method allows the fracture to be entered remotely and repositioned in a closed manner, which is least disturbing to the delicate blood supply of the proximal radius. In all cases, increased invasiveness in the treatment of fractures also appears to result in poorer functional outcomes. Other surgical approaches, such as transarticular Witt's K-wire, internal K-wire, and mini-plate fixation, are associated with poor results, as they either negatively affect the periosteal blood supply or cause damage to the elbow joint [21]. However, when consolidation is completed, the alignment of the proximal radius and the fracture type are less important for evaluating the range of elbow movement. Nevertheless, the extent of radius head deformation is crucial [22]. Once again, closed reduction is the key point for avoiding possible perfusion deficits and healing delays. In completely dislocated fractures, a percutaneously inserted K-wire can manipulate the radial head using a joy-stick-like technique, thus enabling a closed reduction and subsequent ESIN osteosynthesis [23, 24]. Some comparative studies have not shown significant differences in the results after closed and open reduction, and this supports the hypothesis that fracture type and initial degree of dislocation are the most important elements for determining functional outcomes [25]. In contrast, most authors observed a correlation of open reduction with a high rate of complications, such as myositis ossificans [26], synostosis [27], and pseudarthrosis [7]. One of the most feared complications is avascular necrosis of the radial head and nonunion [7, 20], and this sometimes requires radial head resection as an ultimate ratio to restore at least a limited pro-/supination. To prevent necrosis in cases where open reduction is unavoidable, a two-implant ESIN technique enables a frisbee-like clamping of the radial head and is, to the best of our knowledge, reported here for the first time (Fig. 3). This procedure prevents the moving or sintering of the radial head following complete disruption of the nutritive vessels, and thus enables a delayed consolidation despite the risk of dislocation in this condition. In this study, no one used ESIN exclusively as an instrument for reduction and pulled the nail out immediately thereafter. This idea is based on the theory that the radial head, replaced between the metaphysis and the capitellum, is sufficiently fixed by the surroundings, making the nail dispensable [28]. The authors of this article believe that the second anesthesia for metal removal is less burdensome for the patient as opposed to risking secondary dislocation with subsequent surgical interventions. Resorbable implants may further reduce the invasiveness of the treatment by eliminating the need for metal removal. The implants might also further reduce the rate of typical ESIN complications (e.g., irritation of soft tissues by nail ends) [29, 30]. Whether these implants also offer the desired biomechanical properties (e.g., sharpened nail ends for better reduction) must be demonstrated in focused long-term studies.

**Fig. 4:**
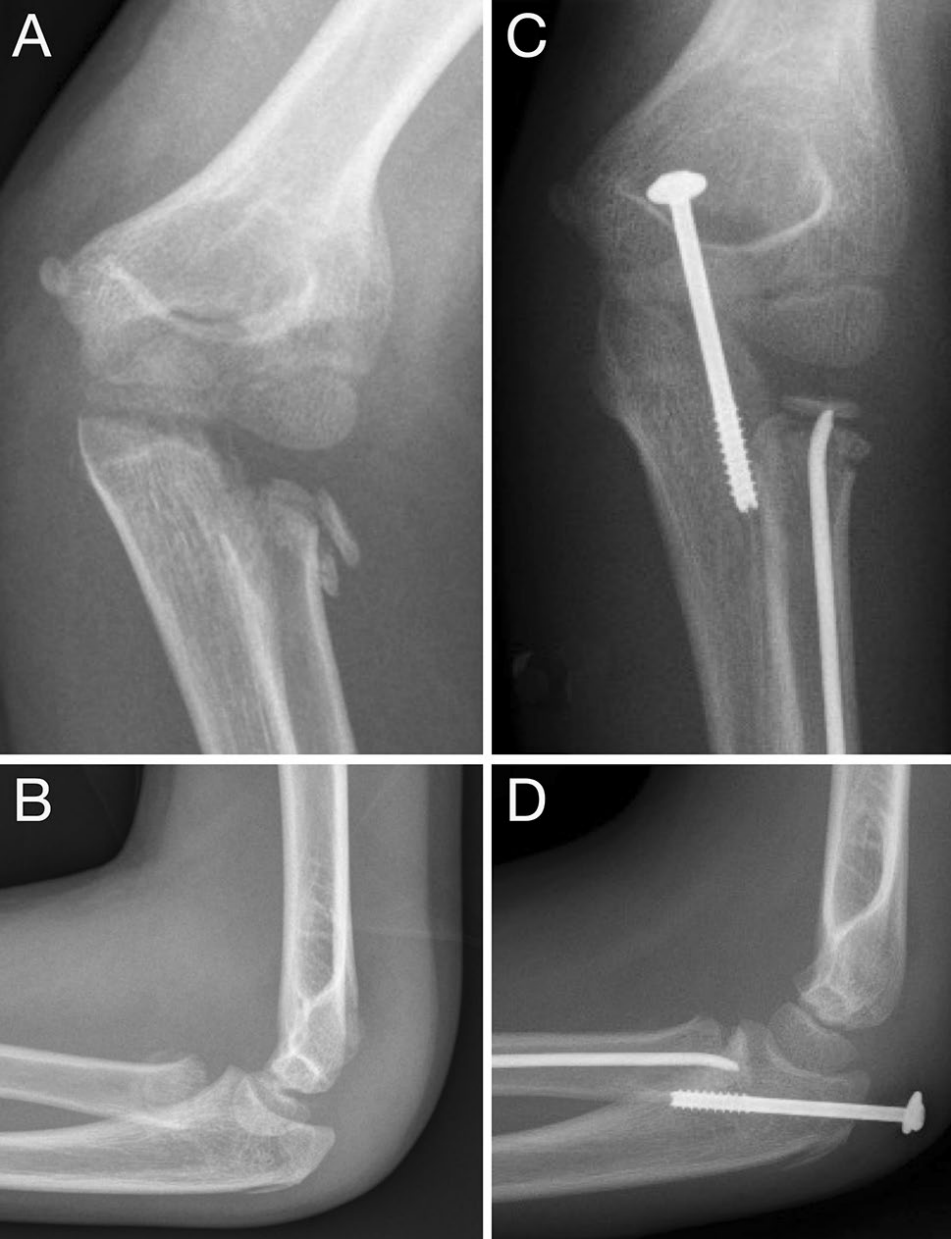
ESIN osteosynthesis in an eight-year-old boy with a Salter–Harris II radial neck fracture (**a**, **b**). A sharpened 2.0 mm titanium nail was used for closed reduction and an additional intraarticular olecranon fracture was treated using a 4.0 mm lag screw to allow early elbow mobilization (**c**, **d**)

Overall, we observe very good results in our patients. However, we note that a large proportion of conservative fractures were included and therefore good results were expected here. It remains unclear, however, as to why completely dislocated fractures also produced excellent results. One explanation is that movement restrictions were treated with a radial head resection in one case, which initially yielded good results. Here, however, it is not conclusively certain—despite a significantly (*p* = 0.008) longer follow-up period in patients with severe injuries—whether ossifications are going to cause other problems in the future (Fig. 4). Another limitation of this study is its retrospective design, therefore cannot allow for conclusions on the aspect of overtreatment. Furthermore, the number of cases is rather small when considering the different subgroups. RHFs also represent a rarity in this study. Consequently, multicenter research or a clinical registry is needed to adequately comment on specific questions.

## Conclusion

In summary, three main factors have to be considered when treating fractures of the proximal radius. The potential of growth-related correction must be included in any therapeutic decision-making process to prevent overtreatment. Every measure, whether conservative or surgical, must be as non-invasive as possible to protect the delicate blood supply of the region. This also applies to follow-up treatment, wherein early functional movement is beneficial and traumatizing physiotherapy is counterproductive. The consistently good treatment results may be individually worsened by additional fractures or accompanying elbow dislocations, which occur frequently in proximal radius fractures.
